# Clinical outcomes of posterior scleral reinforcement in Chinese high myopia children

**DOI:** 10.1038/s41598-024-67078-7

**Published:** 2024-07-17

**Authors:** Haiyun Ye, Ruizhi Tang, Wangyi Fang, Yue Di, Tong Qiao

**Affiliations:** 1 Department of Ophthalmology, Shanghai Children’s Hospital, School of medicine, Shanghai Jiao Tong University, No. 355 Luding Road, Shanghai, 200062 China; 2 Ghent University Centre for X-Ray Tomography (UGCT), Proeftuinstraat 86/N12, 9000 Ghent, Belgium

**Keywords:** Posterior scleral reinforcement, High myopia, Angiography optical coherence tomography (angio-OCT), Axial length, Paediatric research, Eye abnormalities

## Abstract

We aim to observe the posterior scleral reinforcement (PSR) clinical outcomes of children with high myopia and analyze the retinal vessel alteration before and after PSR by using angiography optical coherence tomography (angio-OCT). Fifty-six pediatric participants (112 eyes) clinically diagnosed high myopia were recruited and were treated by PSR in Shanghai Children’s Hospital from June 1, 2021 to May 1, 2023. The average age ranged from 5.42 to 14.83 years (mean 8.83 years) and mean follow up duration was 8.7 months (3–24 months). The axial length (AL) was significantly shortened after PSR (*p* < 0.05). The spherical equivalent (SE) and the best-corrected visual acuity (BCVA) were also improved without severe rejection in the follow-up. Compared with baseline, angio-OCT parafoveal vessel indices including vascular area density (VAD) and vascular skeleton density (VSD) on the superficial capillary plexus layer (SCPL), as well as VAD and vessel perimeter index (VPI) on the deep capillary plexus layer (DCPL), were significantly increased after PSR surgery (*p* < 0.05). VPI on the SCPL, vascular diameter index (VDI) and VSD on the DCPL were also improved without statistical difference after PSR. The VSD on SCPL, VAD on DCPL of the right eyes and the VPI on SCPL of the left eyes were significantly increased after PSR (*p* < 0.05). PSR surgery can shorten the AL and can stable BCVA and SE in high myopia children. The angio-OCT parameters indicated that the retinal microcirculation supply was significantly improved after PSR.

## Introduction

High myopia, defined as refractive error of − 6.00 diopters (D) or worse, is the most frequent cause of children and teenagers’ visual impairment in Asian countries^1^. High myopic eyeballs expanded globally, especially in axial length (AL)^2^. With the AL elongation, high myopia can cause many ocular complications in posterior segment, such as retinal thinning, degeneration, myopic maculopathy, detachment, posterior staphyloma, choroid neovascularization, and eventually lead to non-reversible visual impairment^3^. Previous literature had suggested that eyeball radius increase in high myopia eyes leaded to the retinal blood circulation reduction. And then, a prolonged period of the fundus blood flow decrease resulted in the release of vascular endothelial growth factor (VEGF)^4,5^. Posterior scleral reinforcement (PSR) surgery was first proposed by Shevelev in 1930 and modified continuously during the past decades. It was clinically applied to prevent further vision loss by thickening the sclera over the posterior pole and to slow the AL elongation. A recent study revealed PSR may maintain the microcirculation of eyes with posterior staphyloma and thereby stabilize the best-corrected visual acuity (BCVA)^6^. Furthermore, many researchers indicated that PSR can promote the growth of micro-vessels and increase the blood flow in posterior pole^7–9^. We aim to observe PSR surgery clinical outcomes by analyzing the retinal vessel alteration in pediatric patients with high myopia.

## Materials and methods

### High myopia patients and PSR surgical procedures

High myopia children (56 pediatric participants, 112 eyes, 39 males and 17 females) were recruited and were treated by PSR in Shanghai Children’s Hospital from June 1, 2021 to Oct 1, 2023 in our study. The average age ranged from 5.42 to 14.83 years (mean 8.83 years) and mean follow up duration was 8.7 months (3–24 months). The study was approved by the Medical Ethics Committee of Shanghai Children’s Hospital and conducted in accordance with the Declaration of Helsinki tenets for research involving human subjects. Informed consent was obtained from all included children after a thorough discussion about both the desired positive outcomes and the potential adverse events of the PSR procedure. All children underwent comprehensive ophthalmologic examinations, included BCVA, intraocular pressure, AL (IOL Master, Carl Zeiss Meditec, Dublin, CA, USA), refractive error, slit-lamp examination, and fundus examinations, angiography optical coherence tomography (angio-OCT), B Scan at baseline and postoperative follow-up visit. The BCVA was measured with the Snellen acuity test and were converted to LogMAR for statistical analysis. Refractive error data were presented as the spherical equivalent (SE).

High myopia children were identified and included in our study according to following criteria: progressive high myopia defined as myopic spherical equivalent refraction (SE) ≥ 6.0 diopters (D) after mydriasis and optometry, with an annual increase of SE ≥ 1.0 D, AL > 25 mm for children under 8 years old, the SE was ≥ 7.0 D and the AL was ≥ 26 mm (8–12 years old), the SE was ≥ 8.0 D and the AL was ≥ 27 mm (12 and 18 years old)^9,10^. Corneal and lens-derived non-axial myopia, eye diseases with visual function impairment (e.g., nystagmus, glaucoma, cataract, retinal detachment, macular or peripheral retinopathy, etc.), systemic disease affecting eye health, history of eye trauma or surgeries (e.g., refractive surgery, scleral buckling, vitrectomy, etc.), low examination compliance children were excluded in our study. In addition, high myopia children accompanied by nystagmus or strabismus often had low coordination in OCTA due to poor fixation ability. Despite they had obvious improvement in visual acuity after PSR, we exclude them in our study.

**Figure 1:**
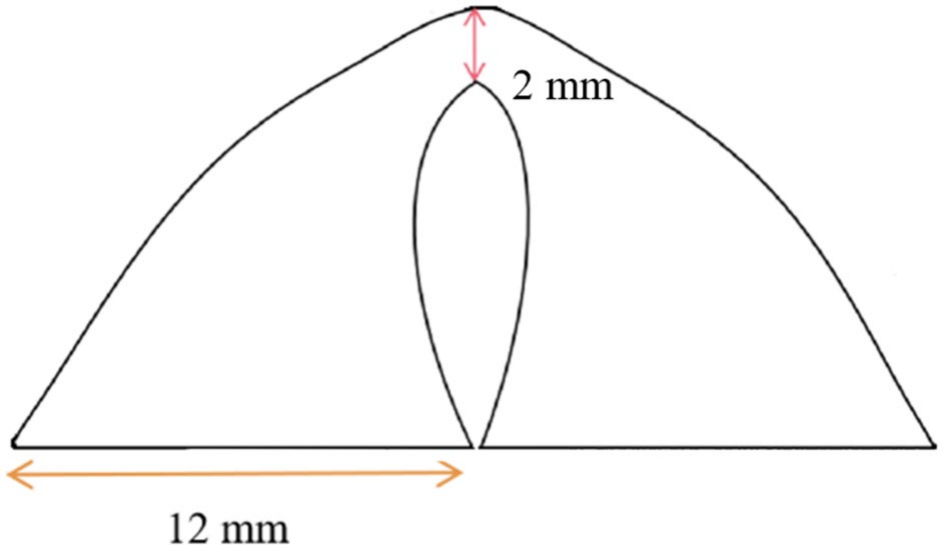
Absorbable dural patch was a double quarter-round pedicled implant with a radius of 12 mm, which was modified by Dr. Qiao.

**Figure 2:**
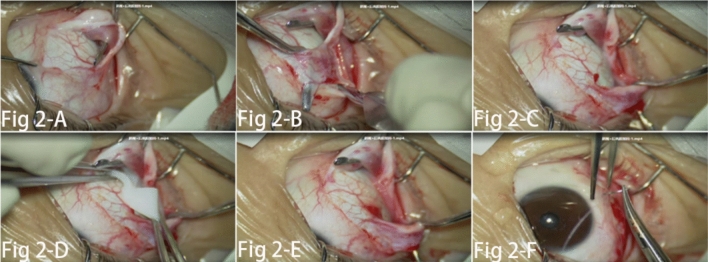
(**A**) showed conjunctiva incision was made at a 2 mm-distance from the temporal-inferior corneal limbus; (**B** and **C**) showed the exposed the lateral rectus muscle and the inferior oblique muscle separately; (**D**) showed the two halves of the patch were successively slid under the lateral rectus muscle and the inferior oblique muscle separately; (**E** and **F**) showed the patch was set flat on the posterior sclera surface by fixing the patch middle pedicle on the sclera between the two muscles with 8–0 absorbable sutures.

All surgeries were performed by Dr Qiao with 30 years experience in ophthalmic microscope surgery. The implant material used in this study was absorbable dural patch from Yantai Zhenghai Biotechnology Co., LTD (Shandong, China) and approved by the China Food and Drug Administration. Absorbable dural patch was a double quarter-round pedicled implant with a radius of 12 mm, which was modified by Dr. Qiao (Fig. 1). Under endotracheal intubation and general anesthesia as well as routine disinfection and draping, a fan-shaped conjunctiva incision about 100° was made at a 2 mm-distance from the temporal-inferior corneal limbus. After the subconjunctiva tissue was separated, expose the lateral rectus muscle and the inferior oblique muscle separately. With subconjunctival fascia was carefully separated, the operator pulled apart the inferior oblique and lateral rectus muscles by the strabismus hook, then the two halves of the patch were successively slid under the two muscles separately. By fixing the patch middle pedicle on the sclera between the two muscles with 8–0 absorbable sutures, the patch was set flat on the posterior sclera surface, which corresponding to the macular area of the retina. Closed the bulbar conjunctival incision with the 8–0 absorbable sutures (Fig. 2).

### Angio-OCT and data statistical analysis

**Figure 3:**
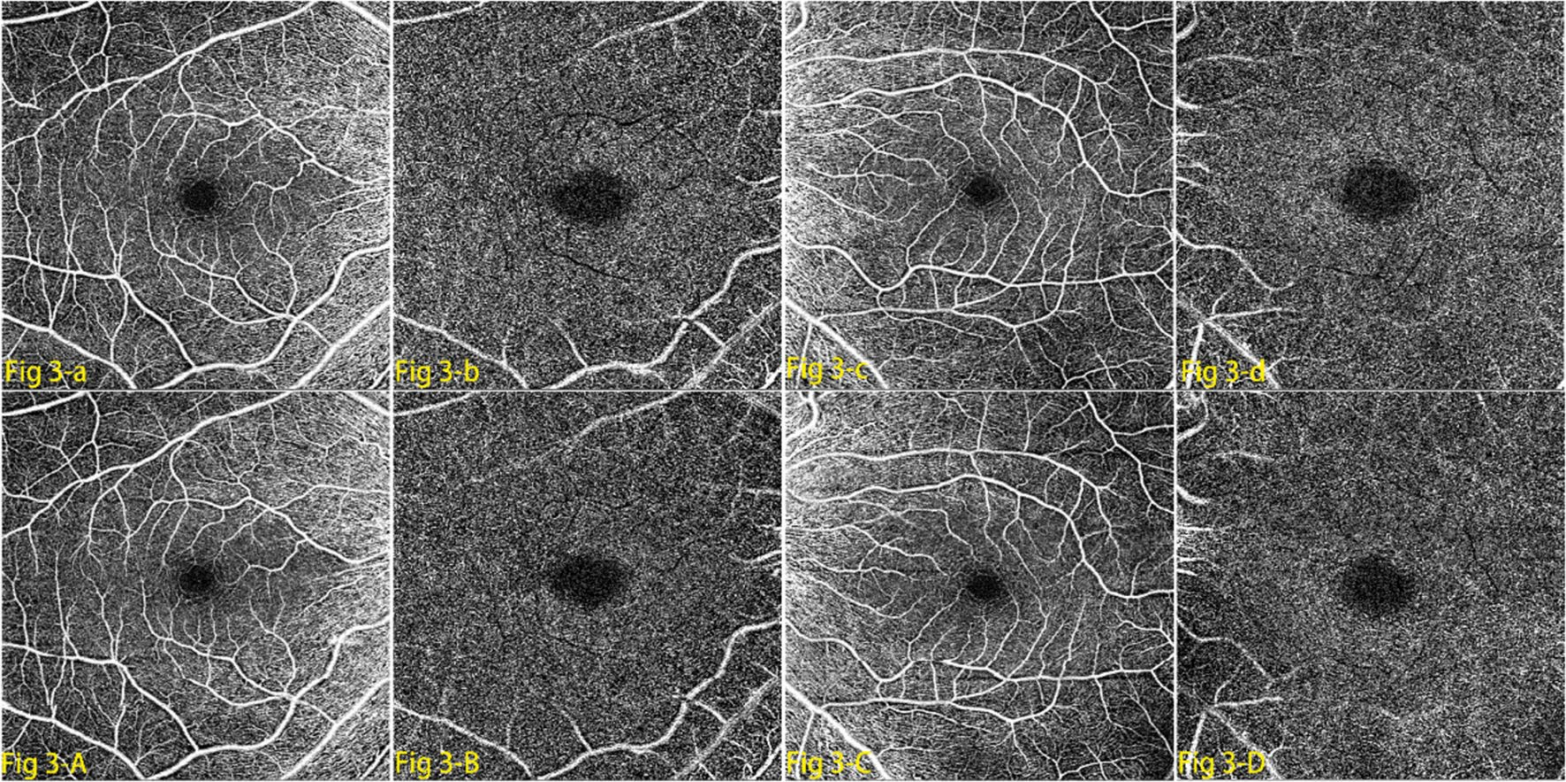
Angiography optical coherence tomography (angio-OCT) images in superfcial capillary plexus layer (SCPL) and deep capillary plexus layer (DCPL) before and after posterior scleral reinforcement (PSR) surgery in both eyes of the same patient. (**a**)–(**d**) showed the SCPL and DCPL in the right eye and the left eye before PSR respectively. (**A**)–(**D**) showed the corresponding position after PSR in the same children.

Angio-OCT images of 6 mm × 6 mm were scanned by Zeiss Cirrus 5000-HD-OCT on the parafoveal areas of the observed eyes (Fig. 3). Effective signal strength index value was set ≥ 8 (range 0 ~ 10). Any image with blur, motion artifact and/or insufficient image quality was excluded. In our research, the parafoveal area was set as an annular capillary-rich area outside the central foveal avascular zone (with an inner diameter of 1 mm and an outer diameter of 3 mm). The retinal vascular density is determined as the percentage area of vessels in the certain segmented areas. As algorithm of the vascular indices described previously, we translated the corresponding imaging processing algorithms and statistics analyzing procedures into Python code. The digital angio-OCT images were loaded and quantitative measurements including vascular diameter index (VDI), vascular skeleton density (VSD), vascular area density (VAD), as well as vessel perimeter index (VPI) on the SCPL and DCPL (superficial and deep capillary plexus layer) were calculated and analyzed before and after PSR. To ensure the reproducibility of the angio-OCT measurements, all subjects were scanned more than three times between 9 and 11 a.m and the images with signal strength index value higher than 8 were collected and the highest quality detection image were chosen in analysis process.

All values are expressed as the mean ± standard deviation (SD). SPSS statistics version 27 (Chicago, IL, USA) was used to analyze the data. The level of significance adopted in the present study was *p* value < 0.05. The paired samples T test was applied to assess the differences pre- and post-PSR surgery between fellow eyes. Correlation analysis between the reduction in AL and retinal vascular parameters were analyzed by Pearson analysis.

### Ethical approval

This project was approved by the Institutional Ethical Review Board of the Shanghai Children’s Hospital that followed the ethical standards of the Helsinki declaration and the corresponding amendments (Approval No: 2022R015-F02). Informed consent was obtained from all the individual participants’ parents or legal guardians included in the study.

## Results

The age of enrolled children ranged from 5.42 to 14.83 years (8.83 ± 2.50) and height ranged from 110 to 174 cm (137.82 ± 17.59) with mean follow up duration of 8.7 months (3–24 months). The grafts were in place during the postoperative follow up without infection, severe rejection, or peripheral retinopathy. The AL (ranged from 25.04 to 29.78 pre-PSR) was significantly shortened after PSR surgery (*p* < 0.05) in the period of follow-up. Post-PSR operation, though without statistical difference, the SE and BCVA were also improved. The observational indexes before and after PSR surgery were shown in Table 1.

**Table 1:** The observational data and basic measurements of participants before and after PSR surgery.

	Pre-PSR	Post-PSR	Mean	SD	*t*	*p*
AL (mm)	26.78 ± 1.26	26.10 ± 0.61	0.68	1.65	4.36	0.00*
BCVA(LogMAR)	0.36 ± 0.31	0.32 ± 0.29	0.04	0.42	1.02	0.31
SE	− 10.17 ± 2.77	− 9.50 ± 2.79	− 0.67	3.91	− 1.81	0.07

PSR, posterior scleral reinforcement; mean, mean differences; SD, standard deviation; AL, axial length; BCVA, best-corrected visual acuity; SE, spherical equivalent.

*Significant statistical difference, *p* < 0.05.

Significant values are in bold.

Compared with baseline, angio-OCT parafoveal vessel indices including VAD and VSD on the SCPL, as well as VAD and VPI on the DCPL, were significantly increased after PSR surgery (*p* < 0.05; Table 2). Though without statistical difference, VPI on the SCPL, VDI and VSD on the DCPL were also improved after PSR surgery (Table 2).Detailed analysis was performed in right and left eyes separately. The VSD on SCPL, VAD on DCPL of the right eyes and the VPI on SCPL of the left eyes were significantly increased after PSR (*p* < 0.05; Table 3). However, the differences of VDI and VAD indices in the SCPL/DCPL did not show statistically significant change.

**Table 2:** Differences in parafoveal SCPL and DCPL before and after PSR surgery by angio-OCT.

		Pre-PSR	Post-PSR	Mean	SD	*t*	*p*
SCPL	VDI	3.58 ± 0.11	3.57 ± 0.09	0.01	0.11	0.70	0.48
	VAD	0.47 ± 0.03	0.47 ± 0.02	0.00	0.02	− 2.15	0.03*
	VSD	0.13 ± 0.01	0.14 ± 0.01	− 0.01	0.02	− 3.25	0.00*
	VPI	0.26 ± 0.01	0.27 ± 0.02	0.00	0.02	− 1.41	0.16
DCPL	VDI	3.58 ± 0.11	3.60 ± 0.08	− 0.02	0.10	− 1.91	0.06
	VAD	0.47 ± 0.03	0.49 ± 0.06	− 0.02	0.05	− 3.86	0.00*
	VSD	0.13 ± 0.01	0.13 ± 0.01	0.00	0.02	− 0.24	0.81
	VPI	0.26 ± 0.01	0.27 ± 0.02	− 0.01	0.02	− 2.80	0.01*

SCPL, superficial capillary plexus layer; DCPL, deep capillary plexus layer; PSR, posterior scleral reinforcement; angio-OCT, angiography optical coherence tomography; SD, standard deviation; VDI, vessel diameter index; VAD, vessel area density; VSD, vessel skeleton density; VPI, vessel perimeter index.

*Significant statistical difference, *p* < 0.05.

Significant values are in bold.

**Table 3:** Monocular differences in parafoveal SCPL and DCPL before and after PSR surgery by angio-OCT.

			Pre-PSR	Post-PSR	Mean	SD	*t*	*p*
SCPL	OD	VDI	3.56 ± 0.10	3.58 ± 0.11	− 0.02	0.10	− 1.23	0.22
		VAD	0.46 ± 0.03	0.47 ± 0.02	0.00	0.02	− 1.78	0.08
		VSD	0.13 ± 0.01	0.14 ± 0.01	− 0.01	0.02	− 3.71	0.00*
		VPI	0.26 ± 0.01	0.27 ± 0.02	0.00	0.03	− 0.68	0.50
	OS	VDI	3.59 ± 0.11	3.56 ± 0.06	0.03	0.12	1.99	0.05
		VAD	0.47 ± 0.02	0.47 ± 0.02	0.00	0.02	− 1.30	0.20
		VSD	0.13 ± 0.01	0.13 ± 0.02	0.00	0.02	− 0.99	0.33
		VPI	0.26 ± 0.01	0.27 ± 0.01	0.00	0.01	− 2.23	0.03*
DCPL	OD	VDI	3.57 ± 0.08	3.58 ± 0.08	− 0.01	0.07	− 1.18	0.24
		VAD	0.47 ± 0.02	0.51 ± 0.06	− 0.03	0.07	− 3.88	0.00*
		VSD	0.13 ± 0.01	0.13 ± 0.01	0.00	0.01	1.01	0.32
		VPI	0.27 ± 0.01	0.27 ± 0.01	− 0.01	0.01	− 3.51	0.00*
	OS	VDI	3.59 ± 0.14	3.61 ± 0.08	− 0.02	0.12	− 1.50	0.14
		VAD	0.46 ± 0.04	0.47 ± 0.04	0.00	0.03	− 1.11	0.27
		VSD	0.13 ± 0.01	0.13 ± 0.01	0.00	0.02	− 0.81	0.42
		VPI	0.26 ± 0.02	0.27 ± 0.02	0.00	0.03	− 1.32	0.19

SCPL, superficial capillary plexus layer; DCPL, deep capillary plexus layer; PSR, posterior scleral reinforcement; angio-OCT, angiography optical coherence tomography; SD, standard deviation; VDI, vessel diameter index; VAD, vessel area density; VSD, vessel skeleton density; VPI, vessel perimeter index.

*Significant statistical difference, *p* < 0.05.

Significant values are in bold.

The correlation coefficient between the AL reduction and the improvement of retinal vascular parameters (VDI and VSD in SCPL as well as VAD in DCPL) showed statistical significance (*p* value respectively 0.03, 0.01, 0.00). However, VAD and VPI in SCPL, VDI, VSD, VPI in DCPL changes did not show correlation with the AL improvement.

## Discussion

Compared with simple myopia (which can achieve good vision through proper optical correction), pathological myopia often associated with poor vision, as well as fast deepened myopia degree, and was prone to fundus complications. High myopia was proved to be caused by a series of complex combination with environmental and genetic factors. Parents’ refractive status as major genetic factor and time spent outdoors as environmental parameter played important roles in the development of high myopia. Abnormal structural alteration of the collagen proteins lead to degenerative changes in the retina (retinal thinning), choroid, and sclera (weakening), which were important mechanisms of progressive axial elongation of the eyeball in high myopic children^11,12^. Currently, it has been suggested that amacrine cells of the retina in the molecular signaling chain regulateed chorioretinal changes, and these alterations may induce poor retinal blood circulation, vascular endothelial growth factor (VEGF) expression, CNV development, posterior pole deformation and staphyloma^11,13^. Thus, we supposed that the earlier the high myopia onset, the more uncontrollable of the retina-related complications appeared. We considered that, during the body growth and development, the in-time intervene on the excessive growth of the eyeball will play a pivotal role in the vision prognosis as well as the prevention and treatment of retinal diseases in high myopia children. Therefore, PSR, which was commonly performed to strengthening the sclera in order to control myopia progression, had been considered as an effective approach to delay the AL increase in progressive high myopia, especially in children^14,15^. In our study, the AL was shortened (*p* < 0.05), the SE and BCVA were improved after PSR surgery in the short term follow-up. According to the results, we supposed that PSR surgery can improve the progression of children high myopia to some extent. However, as children grownup, we cannot predict how long the effect will maintain due to the limited observation period.

Many researchers reported that many graft materials had been used as enhancement patches in PSR, including the scleral buckle, autologous or homologous fascia lata, human sclera, dura, and bovine pericardium. With good biocompatibility and sufficient tensile strength, we chose the absorbable dural patch and covered the weak posterior pole of the sclera with minimal surgical procedures and tissue interference. The PSR performance can be improved by the patching material with strong resistance to enzyme degradation and high retaining mechanical strength^16^. In our study, the grafts were in place without infection or severe rejection. Baohong Wen and colleagues adoped the high-resolution 3D magnetic resonance imaging (MRI) to quantitatively analyze the eyeballs shape in high myopia and provide assistance for PSR. They found that the arc length through the apex of the staphyloma from the upper limbus to the lower limbus and the vertical arc length of the staphyloma are important to determine the size and location of the graft during PSR^17^. However, considered that the posterior scleral staphyloma in childhood was not fully formed, the range of the graft used in our study could cover the posterior scleral surface in childhood as well as sedation of MRI examination in children, we applied B scan instead of MRI to observe the posterior scleral situation postoperatively.

Considered the type of refractive error related to the AL and other refractive components (e.g., cornea and lens), it was not sufficient to evaluate the PSR effectiveness only by AL, SE or BCVA^15^. It was reasoned that the eyeball elongation with retinal and choroidal thinning might reduce the oxygen consumption and lead to macular blood circulation decrease. The decrease was prominent in the superficial capillary plexuses and well-correlated with the retinal thickness profiles^2^. Lin and colleagues found that vessel density in SCPL was unaffected in children and adolescents without high myopia^2^. These showed the macular microvascular network alteration may be attributed to the ocular axial elongation that occurs with myopia. Similar results with Li Jing Mo et al. confirmed the macular vasculature perfusion improvement was essential to visual function of pathological mypia^18^. It was thought that PSR could improve the microcirculation within macula by the secondary non-specific inflammatory reaction between the posterior sclera and the reinforcement band^14^. Consistent with the literature^6,19^, our study showed parameters on SCPL/DCPL not only were improved to varying degrees in the angio-OCT after PSR surgery but also statistically correlated with the AL reduction. This would be a very interesting finding that may provide new ideas on reducing the AL could improve the microvascular structure of the retina on treating certain eye diseases related to retinal blood vessels. Our results suggested that the graft apical pressure at the posterior pole may maintain the curvation and resist the vascular density damage caused by ocular expansion in high myopia after PSR operation. Furthermore, angio-OCT might be a significant and sensitive index in observing the myopia progression. Potential limitations of our study should be mentioned. The absorbable dural patch dimensions in our study were not designed individually according to the curvature of the ocular posterior pole, and the fixation degree could not be quantified. The mechanism of blood flow dencity reation on PSR and how it contributed to the high myopia development remain unclear. In this study, we performed PSR in both eyes of the patients, without a comparison of natural progression in the fellow eye. Further well-designed studies are needed to determine the long-term safety and efficacy of PSR. Besides, we analyzed the binocular gap in results maybe related with right-handed and writing habits in Chinese children. However, there were limited clinical studies on the relationship between monocular high myopia and writing habits as well as pathogenic genes. Thoug the unilateral high myopia patients were not observed in our research, we will follow up and improve it in the future long-term follow-up and hope for providing a sufficient analysis. In the futrure, the efficacy of PSR in unilateral high myopia children who have rapid progression and how PSR maintain or improve perfusion in high myopia patients with needs further research to evaluate.

## Data Availability

The datasets used during the current study are available from the corresponding author upon request.
